# Comparison of clinical outcomes of Ibutilide-guided cardioversion and direct current synchronized cardioversion after radiofrequency ablation of persistent atrial fibrillation

**DOI:** 10.3389/fcvm.2023.1141698

**Published:** 2023-11-06

**Authors:** Xing Liu, Yan He, Chun Gui, Weiming Wen, Zhiyuan Jiang, Guoqiang Zhong, Mingxing Wu

**Affiliations:** ^1^ Department of Cardiology, Xiangtan Central Hospital, Xiangtan, China; ^2^ Department of Cardiology, The First Affiliated Hospital of Guangxi Medical University, Nanning, China

**Keywords:** cardioversion, ibutilide, persistent atrial fibrillation, recurrence, spontaneous conversion

## Abstract

**Backgroup:**

Ibutilide has already been used for cardioversion of persistent atrial fibrillation (PsAF) after radiofrequency catheter ablation (RFCA). The purpose of this study was to determine the effect of Ibutilide-guided cardioversion on clinical outcomes after individualized ablation of PsAF.

**Methods:**

From October 2020 to September 2021, consecutive patients with PsAF accepted for RFCA were prospectively enrolled. After individualized ablation including pulmonary vein isolation plus left atrial roof line ablation and personalized linear ablation based on left atrial low-voltage zones, patients were divided into the spontaneous conversion (SCV) group, direct current synchronized cardioversion (DCC) group and Ibutilide group according to different cardioversion types during ablation. The rates of freedom from atrial tachyarrhythmia (ATT) among the three groups were evaluated after follow-up.

**Results:**

In this study, 110 patients were enrolled, including 12 patients with SCV, 50 patients receiving DCC and 48 patients receiving Ibutilide cardioversion after individualized ablation. Among the three groups, the SCV group had shorter AF duration {12 months [interquartile range (IQR) 12–16], *P* = 0.042} and smaller left atrial diameter (LAD) [35 mm (IQR: 33–42), *P* = 0.023]. A 12-month freedom from ATT rate was 83.3% in SCV group, 69.4% in DCC group, and 79.2% in Ibutilide group, respectively (Log-rank, *P* = 0.745). During the follow-up [17 months (IQR: 15–19)], the rate of freedom from ATT of SCV group (83.3%), and Ibutilide group (72.9%) were both higher than that of DCC group (53.1%, *P* = 0.042). Moreover, Kaplan–Meier analysis showed a significantly higher sinus rhythm (SR) maintenance in Ibutilide group than in DCC group (Log-rank, *P* = 0.041). After adjusting for risk factors of AF recurrence, the hazard ratio for AF recurrence of the DCC group with reference to the Ibutilide group was 4.10 [95% confidence interval (CI) (1.87–8.98), *P* < 0.001]. Furthermore, subgroup analysis showed that freedom from ATT rate in effective Ibutilide subgroup was significantly higher than noneffective Ibutilide subgroup (Log-rank, *P* < 0.001).

**Conclusion:**

For the treatment of the patients with PsAF, Ibutilide-guided cardioversion after individualized RFCA may be benefit for maintenance of SR compared to conventional DCC, especially for the patients who are effective for administration of Ibutilide.

## Introduction

Atrial fibrillation (AF) is the most frequently occurring cardiac arrhythmia and its presence increases the risk of embolic stroke, heart failure, cognitive impairment and mortality (1). Despite the fact that radiofrequency catheter ablation (RFCA) remarkably improves the quality of life and reduces the risk of mortality for patients with AF (2), the long-term efficacy of RFCA in the patients with persistent AF (PsAF) remains discouraging (3). Current guidelines have identified pulmonary vein isolation (PVI) as the cornerstone of catheter-based treatment for AF, and PVI alone is insufficient to maintain sinus rhythm (SR) in the patients with PsAF (4). Therefore, supplementary ablation strategies, including linear ablation (5), complex fractionated atrial electrogram (CFAE) ablation (5, 6), rotor modulation-guided ablation (7), and substrates ablation (8) have been widely used for ablation of PsAF in recent decades. However, the overall success rate is not significantly improved and the optimal ablation strategy for PsAF remains undetermined. Some studies have shown that pulmonary vein reconnection is closely related to AF recurrence (9, 10), and the gap-related atrial tachyarrhythmia caused by excessive ablation is one of the leading causes for the recurrence of AF after RFCA (11, 12). Therefore, the PVI durability and complete ablation path without gap, due to durable and transmural ablation lesions, are specifically important for RFCA of PsAF.

More durable lesions are produced by ablation guided by ablation index (AI) that combines contact force, power, and time (13). Meanwhile, high power further promotes irreversible tissue damage by increasing the effect of resistive heating (14). Clinical studies have reported that high power shorter duration ablation guided by AI (HPSD-AI) produces durable ablation lesion and may reduce AF recurrence after RFCA (15, 16). On the other hand, direct current synchronized cardioversion (DCC) or Ibutilide has commonly been used for conversion of PsAF after PVI plus extra ablation in some clinical centers for AF. Moreover, Ibutilide could reveal critical sites that are obscured by the failure of SR recovery during ablation in patients with PsAF (17). After intravenous administration with Ibutilide, there were three responses–conversion to SR, or atrial flutter (AFL)/atrial tachycardia (AT), or continuation of AF. The patients with AFL/AT were treated with mapping and targeted ablation and reverted to SR, whereas those who continued to be in AF were electrically cardioverted to SR. However, the clinical outcomes of ablation combined with Ibutilide-guided cardioversion in patients with PsAF remains unclear. Therefore, this study aimed to observe the effect of Ibutilide-guided cardioversion on clinical outcomes in the patients with PsAF, after the guidance of HPSD-AI ablation.

## Methods

### Study participants

Two-center, prospective observational study was conducted at the First Affiliated Hospital of Guangxi Medical University and Xiangtan Central Hospital, and consecutively enrolled the patients with PsAF who underwent their first RFCA according to the expert consensus on catheter ablation for AF (9) between 1 October 2020 and 30 September 2021. AF that is continuously sustained beyond 7 days, including episodes terminated by cardioversion (drugs or electrical cardioversion) after ≥7 days is defined as PsAF, according to 2020 ESC guidelines for the diagnosis and management of atrial fibrillation (4). All enrolled patients underwent RFCA with HPSD-AI ablation model according to our previous study (15). Exclusion criteria included age <20 or ≥85 years, valvular AF, an left atrial (LA) diameter ≥55 mm evaluated by echocardiography, a history of cardiac surgery, contraindication to systemic anticoagulation, or left atrial appendage thrombus. The study was approved by the institutional review board of the two participating hospital, and conducted in accordance with the 1975 Helsinki Ethics Guide. All patients submitted written informed consent. The study was registered at Chinese Clinical Trial Registry (http://www.chictr.org.cn, No.ChiCTR2200066444, Retrospectively registered).

### Study protocol

**Figure 1:**
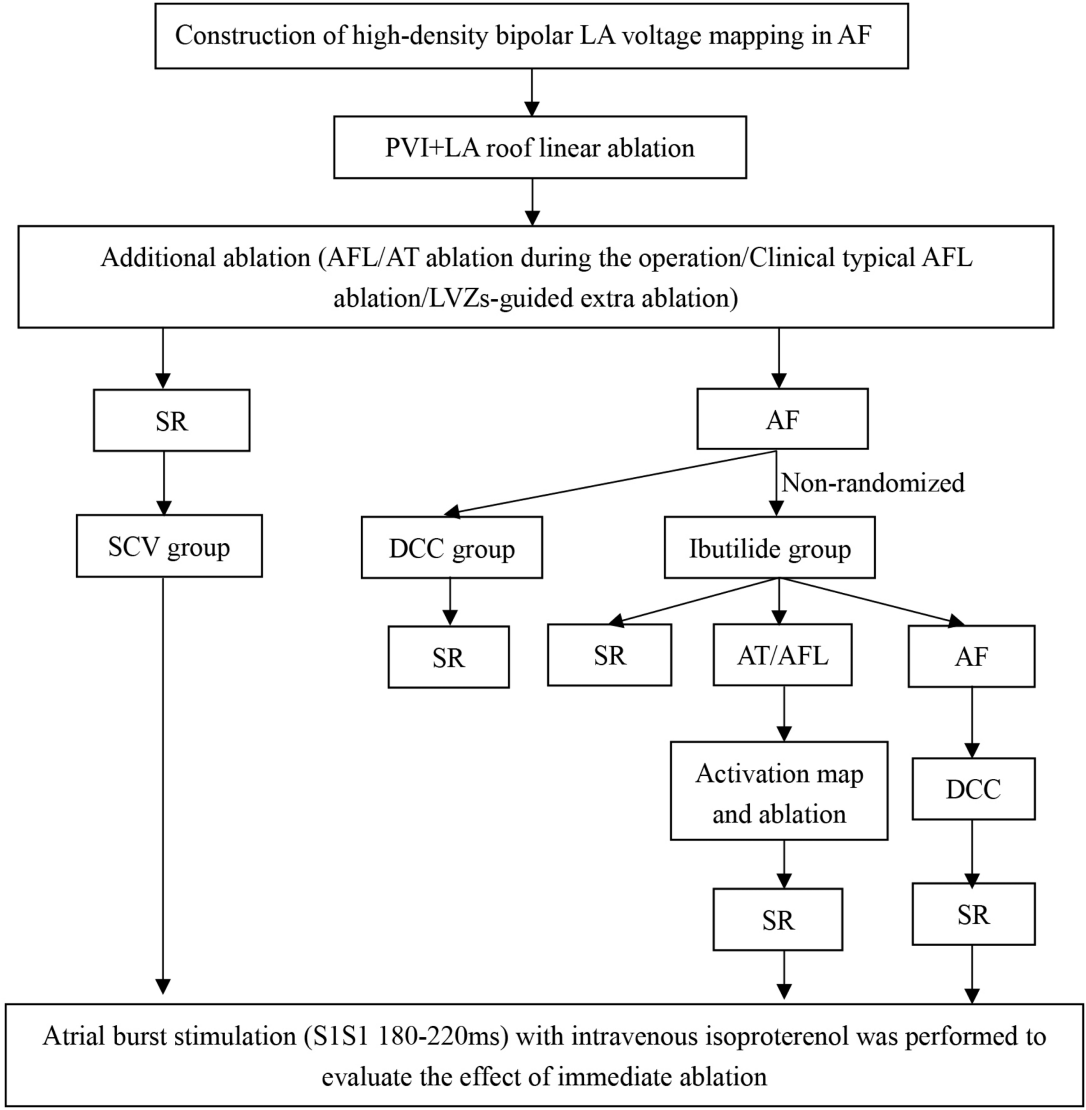
Study design. AF, atrial fibrillation; AFL, atrial flutter; AT, atrial tachycardia; DCC, direct current synchronized cardioversion; LA, left atrial; LVZs, low voltage zones; PVI, pulmonary vein isolation; SCV, spontaneous conversion; SR, sinus rhythm.

**Figure 2:**
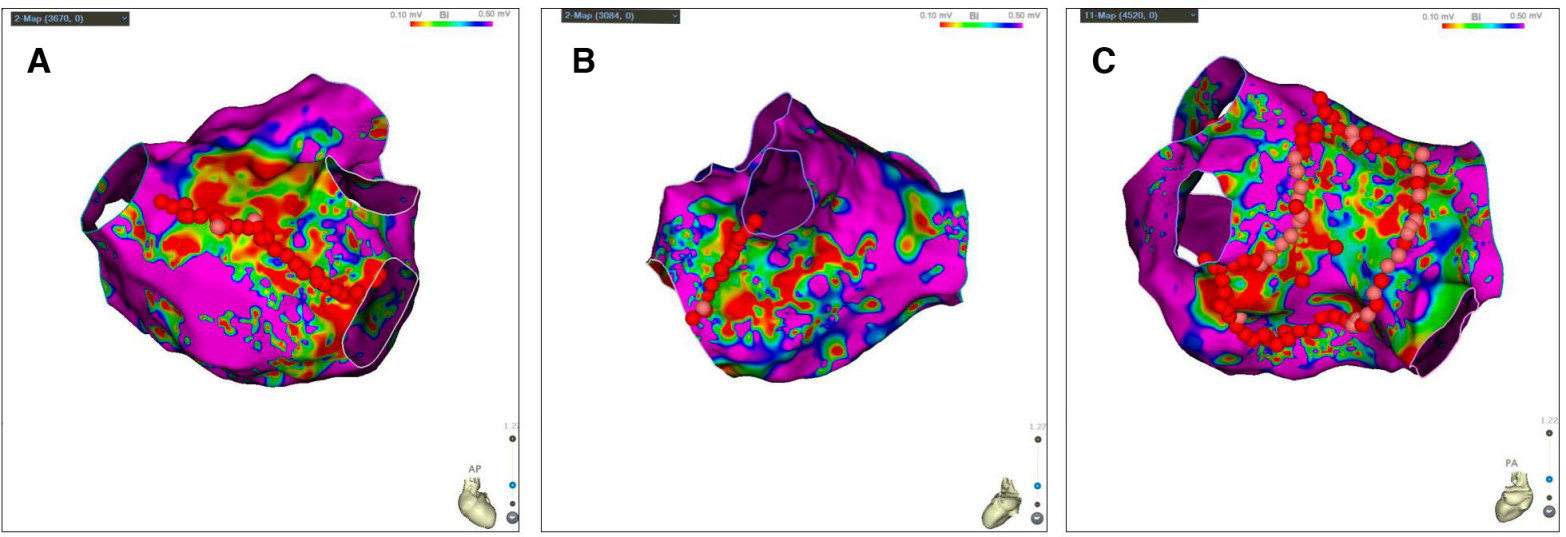
LVZs-guided extra linear ablation. (**A**) LA posterior wall Box ablation if there were sufficient LVA in the LA posterior wall; (**B**) Mitral isthmus ablation if LA isthmus contained significant LVA; (**C**) Anterior linear ablation if LA anterior involved significant LVA. Bipolar voltage <0.5 mV represented the LVZs (non-purple) and bipolar voltage >0.5 mV represented the normal substrate (purple). LA, left atrial; LVZs, low voltage zones; LVA, low-voltage area.

The study design is shown in Figure 1. In order to furthest avoid iatrogenic atrial arrhythmias due to excessive ablation, and to ensure efficient ablation, individualized ablation strategies were adopted for PsAF. At first, all patients underwent PVI and LA roof line ablation after construction of high-density bipolar LA voltage mapping under AF rhythm. Then, additional ablation procedures was performed, such as activation mapping and ablation if AF was converted to AFL or AT during the operation. Additionally, cavotricuspid isthmus linear ablation was executed, if the patients had clinical typical AFL in this study. Furthermore, we also underwent personalized linear ablation based on LA low-voltage zones (LVZs) in LA, including posterior wall box lesion (adding posterior inferior line) if there were sufficient low-voltage area (LVA) in the LA posterior wall, mitral isthmus ablation if it contained significant LVA, and anterior linear ablation if it involved significant LVA. LVZs-guided extra linear ablation is shown in Figure 2. The patients whose AF were successfully terminated after the aforementioned operations, constituted the spontaneous conversion (SCV) group. The patients, whose AF were not terminated by the index ablation strategy, were non-randomly assigned to receive DCC (DCC group) or Ibutilide intravenously (Ibutilide group) based on the operators' assessment for the patient's condition and intraoperative situation. If the patient could not tolerate prolonged process of operation, or had a obviously lengthened corrected QT interval caculated by Bazett's formula (18), the operator would terminate AF of the patient by DCC. Except for these patients, the patinets were assaigned into DCC group or Ibutilide group with 1:1 ratio according the match based on sex, gender,and underlying diseases as soon as possible. In the Ibutidide group, we did not perform further ablation if AF was converted to SR after administration of Ibutilide. If the patient had converted from AF to AFL/AT, activation mapping and targeted ablation were performed to eliminate AFL/AT. If the patients failed to terminate AF to regular rhythm (SR or AFL/AT) after twice administration of Ibutilide, electrical cardioversion would be further conducted. Entrance and exit block of the pulmonary veins and bidirectional conduction block following ablation of any isthmus or ablation line was confirmed after all patients returned to SR. At last, atrial burst stimulation (S1S1 180–220 ms) in the condition where heart rate increased by 20%–30% compared to baseline by intravenous isoproterenol was performed to evaluate the effect of immediate ablation and determined whether further ablation was needed, such as ablation for non-PV triggers inducing AF/AFL/AT. The ablation procedure was terminated if tachycardia could not be induced or could not be maintained.

### LA voltage mapping and catheter ablation procedures

After single transseptal puncture, heparin (100 IU/kg) was administered intravenously with the activated clotting time maintained for 300–350 s. A 20-pole multielectrode catheter (PentaRay Nav Catheter; Biosense Webster) and an open-irrigated ThermoCool SmartTouch Surround Flow (STSF) catheter (Biosense Webster, CA, USA) were used for LA voltage mapping and ablation. LA voltage mapping was performed under AF rhythm. Considering temporal electrogram amplitude variability during AF, each point was manually selected. During LA mapping, the multielectrode catheter was stably attached to the tissue in the same site for at least 5–8 s to assess consistency of intracardiac electrocardiogram patterns to avoid or minimize errors. To ensure highest accuracy of intracardiac electrocardiogram criteria, endocardial voltage maps had a minimum target of 1,500 endocardial points and a maximum point density of 1 mm. LVZs was determined by the presence of ≥3 adjacent points exhibiting voltage <0.5 mV during AF (19–21). The LA surface area was defined as the area of the LA body excluding the LA appendage, PV antrum regions, and mitral valve. LVA was defined as the mean percentage of LVZs to LA surface area. Meanwhile, quantitative assessment of atrial surface area and LVA was performed using standardized software (CARTO 3, Biosense Webster, Diamond Bar, CA). To assess the extent of LVZs, the LA was divided into 6 anatomical regions: (1) anterior excluding left atrial appendage, (2) posterior, (3) roof, (4) inferior, (5) septal, (6) lateral. HPSD-AI ablation mode was high-power (40–50 w) and delivered for ≤20 s until the target ablation lesion index was achieved: AI ≥ 350 in sites on the LA posterior wall and ≥410 in others. All the procedures were performed by experienced specialists.

### Medication management

All antiarrhythmic drugs (AADs) were stopped in patients for at least 5 half-lives, and amiodarone was discontinued for 1 month at least prior to ablation, unless the arrhythmia was uncontrolled according to physician preference. The patients could receive uninterrupted warfarin therapy with INR 2.0–3.0, or the patients who previously treated with novel oral anticoagulants (NOACs) stopped 24 h before the ablation procedure and restarted 6 h afterward. The preoperative electrolyte results of all patients indicated normal blood potassium and magnesium. In the Ibutilide group, Ibutilide 1 mg diluted in 20 ml of 0.9% sodium chloride was administered via a slow intravenous injection for over 10 min. If AF rhythm was still persisted 10 min after the first Ibutilide administration, second dosage was given. Once AF converted to SR, AT, occurrence of ventricular tachycardia, or any adverse reaction, Ibutilide was immediately stopped. Adverse reactions were observed in all patients within 6 h after Ibutilide administration. After the procedure, all patients received AADs (amiodarone or propafenone) for up to 3 months, anticoagulation therapy (warfarin or NOACs) for at least 3 months, and proton pump inhibitors for 6 weeks.

### Data collecting and follow-up

Baseline patient characteristics, 24 h Holter electrocardiogram, transthoracic echocardiography (TTE), transoesophageal echocardiography (TEE), and enhanced cardiac computed tomography were routinely collected at enrollment. Left atrial appendage ostium diameter (LAAOD) that was the maximum opening distance, and left atrial appendage depth (LAAD) that was the longest depth of left atrial appendage were measured at different angles under TEE, respectively. Ablation characteristics such as ablation parameters, fluoroscopy dose, procedure time (defined as the time from sterilization to the end of the procedure), and *P*-wave dispersion (Pd) were recorded after ablation. Pd was defined as the difference between the maximum and minimum *P*-wave duration recorded from the 12 electrocardiogram (ECG) leads in SR after the operation. AF recurrence after ablation was defined as occurrence of atrial tachyarrhythmia (ATT, including AF, AFL or AT) lasting >30 s after the initial 3-month blanking period as per current guidelines (9). All the patients were followed up for at least 12 months after ablation. Twelve lead ECG was regularly performed for the patients 1, 3, 6, 9, and 12 months after RFCA. A 24 h Holter was performed at 6 and 12 months after RFCA. The follow-up was executed every 1–3 months. If the patients experienced symptoms, the additional visits were fulfilled. Simultaneously, the complications such as atrioesophageal fistula, pericardial effusion and ischemic stroke were monitored during follow-up. The follow-up ended until 30 September 2022.

### Study endpoints

The primary endpoint of the study was freedom from recurrence of any ATT (>30 s), after a blanking period of 3 months, at 1 year and at the end of follow-up after the initial ablation procedure. The secondary endpoints were to investigate the independent risk factors related to AF recurrence after RFCA and the assessment of complications of RFCA among the groups at the end of follow-up.

### Statistical analysis

Continuous variables were tested for normality using the Kolmogorov–Smirnov test. Continuous variables in each group were expressed as mean ± standard deviation or as median with interquartile range (IQR) according to the distribution type of the dada, and compared using one-way analysis of variance (ANOVA) or Kruskal–Wallis test, respectively. Categorical data were expressed as frequencies (percentages) and compared with the chi-squared test or Fisher's exact test, as deemed appropriate. The risk factors of ATT recurrence were analyzed by univariate analysis and multivariate Cox regression analysis. The independent predictors of AF recurrence after catheter ablation were determined by multivariate Cox proportional hazard regression. C statistic was performed to assess the discrimination of multivariate Cox model (22). Because of a small size in our study, for further model validation, 1,000 bootstrap samples were used to derive a validation C statistic that would correct for potential model overfitting (22). Multivariate-adjusted Cox models were constructed to determine the relationship between different cardioversion types and AF recurrence at the end of follow-up. The risk factors were expressed as a hazard ratio (HR) with 95% confidence interval (CI) vs. controls. The Kaplan–Meier curve was used to calculate the cumulative freedom from ATT rate after RFCA at one year and at the end of follow-up. *P*-value <0.05 was considered statistically significant. C statistic and bootstrapping for model validation were performed using “survival package” and “boot package” in R software (https://cran.r-project.org/), respectively. All other analyses were performed using IBM SPSS version 26.0 (IBM Corp.) and GraphPad Prism 8 (GraphPad Software, San Diego, California, USA).

## Results

### Participants and baseline characteristics

**Figure 3:**
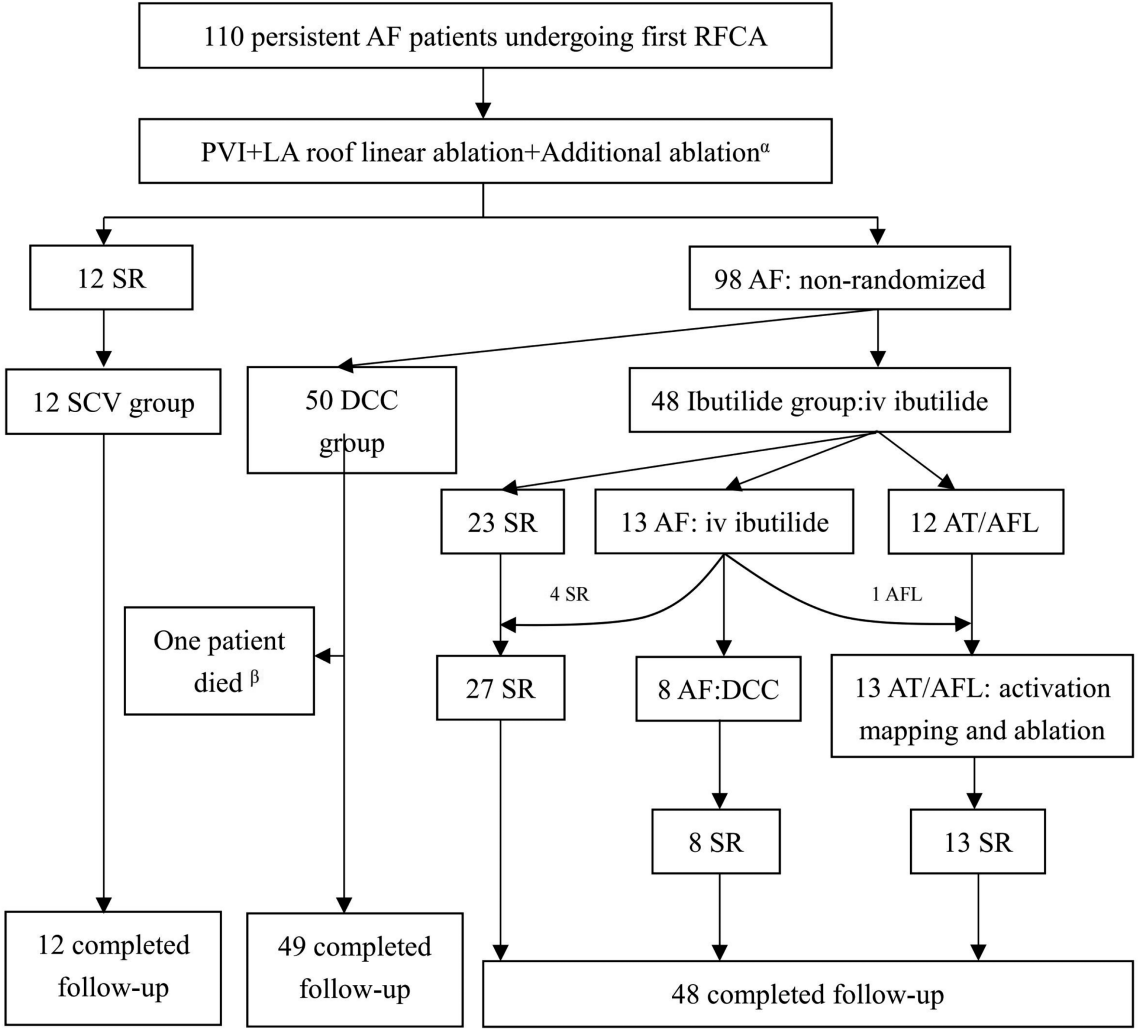
Patients flow of the study. ^α^Additional ablation inclued AFL/AT ablation during the operation, clinical typical AFL ablation, or LVZs-guided extra ablation; ^β^One patient in DCC group died of traffic accident and was lost to follow-up. AF, atrial fibrillation; AFL, atrial flutter; AT, atrial tachycardia; DCC, direct current synchronized cardioversion; LA, left atrial; LVZs, low voltage zones; PVI, pulmonary vein isolation; SCV, spontaneous conversion; SR, sinus rhythm.

A total of 110 patients [median age: 63 years (IQR: 54–66); 63.6% males] were consecutively enrolled. A flow chart depicting the classification of the patients is shown in Figure 3. Twelve patients (10.9%) whose AF was terminated by the index ablation strategy (conversion to AT/AFL in 7 patients and restoring to SR in 5 patients) were included in the SCV group. The 7 AT/AFL, including typical AFL [*n* = 3], roof dependent [*n* = 1], mitral isthmus AFL [*n* = 2], and focal AT from LA posterior wall [*n* = 1], were eliminated by targeted ablation. There were 50 patients in DCC group, and 48 patients in Ibutilide group, respectively. In the Ibutilide group, 27 patients (56.3%) converted to SR (Ibutilide conversion subgroup), 13 patients (27.1%) converted to AT/AFL (AT/AFL subgroup), and the other 8 patients (16.6%) underwent successful DCC (DCC subgroup). The 13 AT/AFL including typical AFL [*n* = 9], mitral isthmus AFL [*n* = 3], focal AT from LA anterior wall [*n* = 1], were successfully terminated by activation mapping and ablation in AT/AFL subgroup. One patient in DCC group died of traffic accident and was lost to follow-up, and 109 patients with PsAF completed follow-up at the end of the study. The median of follow-up duration was 16 months (IQR: 13–18) in SCV group, 17 months (IQR: 15–19) in DCC group and 17 months (IQR: 15–20) in Ibutilide group, and there was no significant difference among the three groups (*P* = 0.387). AF duration was different among the three groups (*P* = 0.042), with the minimum duration in SCV group. The smallest LAD was in SCV group compared with other groups (*P* = 0.023). The other baseline clinical characteristics did not statistically different among the three groups (Table 1).

**Table 1:** Comparison of patient baseline clinical characteristics among SCV group, DCC group and Ibutilide group.

Variable	SCV group (*n* = 12)	DCC group (*n* = 50)	Ibutilide group (*n* = 48)	*P*-value
Demographics				
Age (year)	57 (47, 69)	65 (55, 67)	61 (54, 66)	0.309
Male sex, *n* (%)	9 (75%)	31 (62%)	31 (64.6%)	0.689
BMI (kg/m^2^)	23 (22, 25)	23 (22, 24)	23 (22, 25)	0.942
Smoking, *n* (%)	7 (58.3%)	19 (38%)	21 (43.8%)	0.434
AF duration (month)	12 (12, 16)^a,b^	19 (12, 48)	22 (14, 36)	**0** **.** **042**
CHA_2_DS_2_-VASc score	2 (0, 3)	2 (1, 3)	2 (1, 3)	0.549
Comorbidities				
Hypertension, *n* (%)	7 (58.3%)	23 (46%)	26 (54.2%)	0.622
Diabetes, *n* (%)	1 (8.3%)	7 (14%)	9 (18.8%)	0.605
Coronary heart disease, *n* (%)	2 (16.7%)	10 (20%)	9 (18.8%)	0.962
Stroke, *n* (%)	1 (8.3%)	3 (6%)	3 (6.3%)	0.959
Preoperative medication				
Warfarin, *n* (%)	2 (16.7%)	7 (14%)	7 (14.6%)	0.973
Rivaroxaban, *n* (%)	10 (83.3%)	43 (86%)	41 (85.4%)	0.973
ACEI/ARB, *n* (%)	5 (41.7%)	24 (48%)	15 (31.3%)	0.237
Lipid-lowering drug, *n* (%)	1 (8.3%)	10 (20%)	11 (22.9%)	0.475
Biochemical indicators				
HGB (g/L)	143 (126, 145)	136 (129, 143)	136 (130, 145)	0.839
Scr (umol/L)	64 (58, 92)	71 (61, 94)	72 (59, 83)	0.884
CKMB (U/L)	11 (10, 13)	12 (10, 13)	13 (11, 14)	0.140
CRP (mg/L)	4 (3, 5)	4 (3, 5)	4 (3, 5)	0.957
TTE				
LAD (mm)	35 (33, 42)^a,b^	41 (39, 45)	42 (38, 46)	**0**.**023**
LVEDD (mm)	48 (46, 51)	46 (44, 49)	47 (44, 49)	0.229
LVEF (%)	67 (65, 70)	65 (58, 69)	63 (58, 67)	0.107
TEE				
LAAOD(mm)	25 (23, 25)	25 (23, 26)	25 (24, 26)	0.233
LAAD(mm)	25 (24, 27)	26 (25, 31)	27 (25, 32)	0.250

ACEI/ARB, angiotensin converting enzyme inhibitor or angiotensin receptor blocker; AF, atrial fibrillation; BMI, body mass index; CKMB, creatine kinase isoenzyme; CRP, C-reactive protein; DCC, direct current synchronized cardioversion; HGB, hemoglobin; LAAOD, Left atrial appendage ostium diameter; LAAD, left atrial appendage depth; LAD, left atrial diameter; LVEDD, left ventricular end-diastolic diameter; LVEF, left ventricular ejection fraction; Scr, serum creatinine; SCV, spontaneous conversion; TEE, transesophageal echocardiography; TTE, transthoracic echocardiography.

Bold values represent *P* < 0.05.

^a^ Post hoc comparison *P* < 0.05 vs. DCC group.

^b^ Post hoc comparison *P* < 0.05 vs. Ibutilide group. Values are given as median (interquartile range) or *n* (%).

### Catheter ablation and complications

The details of ablation procedures and complications are shown in Table 2. High density voltage mapping of the LA was performed in all the patients with a mean number of 2,051 points (IQR: 1,999–2,130). A total of 75 patients (68.2%) were found to have LVZs, including 5 patients (41.7%) in SCV group, 36 patients (72%) in DCC group, and 34 patients (70.8%) in Ibutilide group. Although more LVA were observed in the DCC group and the Ibutilide group [0% (IQR: 0–13.5) in the SCV group, 9% (IQR: 0–28) in the DCC group, and 8.5% (IQR: 0–33.5) in the Ibutilide group, respectively], there were not statistically significant differences among the three groups (*P* = 0.157).

**Table 2:** Comparisons of ablation characteristics.

Variable	SCV group (*n* = 12)	DCC group (*n* = 50)	Ibutilide group (*n* = 48)	*P* value
LA mapping points	2,100 (1,999, 2,175)	2,100 (2,000, 2,180)	2,100 (2,000, 2,200)	0.779
Left atrial volume (ml)	119 (114, 134)^a,b^	139 (130, 145)	137 (131, 145)	**0** **.** **009**
LVA (%)	0 (0, 13.5)	9 (0, 28)	8.5 (0, 33.5)	0.157
Low voltage ratio, *n* (%)	5 (41.7)	36 (72)	34 (70.8)	0.128
>40%, *n* (%)	0 (0)	12 (33.3)	12 (35.3)	0.373
10%–40%, *n* (%)	4 (80)	12 (33.3)	9 (26.5)	0.069
<10%, *n* (%)	1 (20)	12 (33.4)	13 (38.2)	0.692
Low voltage distribution in LA				
Anterior wall, *n* (%)	2/5 (40)	16/36 (44.4)	16/34 (47.1)	0.946
Posterior wall, *n* (%)	3/5 (60)	17/36 (47.2)	17/34 (50.0)	0.861
Septum, *n* (%)	1/5 (20)	3/36 (8.3)	5/34 (14.7)	0.607
Inferior wall, *n* (%)	1/5 (20)	10/36 (27.8)	10/34 (29.4)	0.903
Roof, *n* (%)	3/5 (60)	15/36 (41.7)	12/34 (35.3)	0.557
Lateral, *n* (%)	1/5 (20)	5/36 (13.9)	4/34 (11.8)	0.764
First-pass RPVI, *n* (%)	9 (75)	39 (78)	40 (83.3)	0.723
First-pass LPVI, (%)	11 (91.7)	44 (88)	39 (81.3)	0.512
PV ablation parameters				
RPV anterior wall AI	434 (431, 442)	434 (432, 444)	433 (428, 441)	0.453
RPV posterior wall AI	394 (389, 400)	400 (388, 401)	400 (398, 400)	0.236
RPV roof/bottom AI	422 (418, 431)	423 (419, 430)	423 (421, 429)	0.808
LRV ridge AI	466 (457, 479)	466 (457, 477)	460 (455, 470)	0.183
LPV posterior wall AI	388 (381, 390)	388 (378, 390)	388 (385, 399)	0.623
LPV roof/bottom AI	421 (418, 423)	423 (420, 428)	422 (422, 428)	0.091
Contact force (g)	10 (8, 10.5)	11 (10, 11)	11 (10, 12)	0.103
Impedance drop (*Ω*)	10 (9.5, 11)	11 (9, 11)	10 (8.5, 11.5)	0.635
Interlesional distance (mm)	4 (3.9, 4.2)	4 (3.8, 4.2)	4 (3.8, 4.3)	0.854
Ablation line and block rate (%)				
LA roof line	11/12 (91.6)	47/50 (94)	46/48 (95.8)	
LA anterior wall line	–	1/2 (50)	2/3 (66.7)	
LA BOX	2/3 (66.7)	10/13 (76.9)	11/13 (84.6)	
Tricuspid isthmus line	6/6 (100)	10/10 (100)	15/15 (100)	
Mitral isthmus line	1/2 (50)	3/5 (60)	6/8 (75)	
Superior vena cava	1/1 (100)	1/1 (100)	–	
Fluoroscopy dose (mGy)	75 (62, 90)^b^	84 (66, 97)^b^	95 (77, 100)	**0**.**028**
Procedure time (min)	181 (153, 205)^b^	178 (168, 200)^b^	200 (187, 230)	**0**.**001**
Steam pop ratio, *n* (%)	0 (0)	1 (2)	1 (2.1)	0.792
Vasovagal reaction, *n* (%)	1 (8.3)	2 (4)	3 (6.3)	0.597
*P*-wave dispersion (ms)	43 (35, 52)^a,b^	59 (45, 72)	60 (46, 69)	**0**.**027**
Complications, *n* (%)	0 (0)	2 (4)	2 (4.2)	0.624
Antiarrhythmic drugs				
Amiodarone, *n* (%)	9 (75)	45 (90)	44 (91.7)	0.246
Propafenone, *n* (%)	3 (25)	5 (10)	4 (8.3)	0.246

DCC, direct current synchronized cardioversion; LA, left atrial; LPV, left pulmonary vein; LPVI, left pulmonary vein isolation; LVA, low voltage area; RPV, right pulmonary vein; RPVI, right pulmonary vein isolation; SCV, spontaneous conversion.

Bold values represent *P* < 0.05.

^a^ Post hoc comparison *P* < 0.05 vs. DCC group.

^b^ Post hoc comparison *P* < 0.05 vs. Ibutilide group. Values are given as median (interquartile range) or *n* (%).

LA roof line ablation was performed in all the patients, and the bidirectional conduction block rate was 11/12 (91.6%) in SCV group, 47/50 (94%) in DCC group, and 46/48 (95.8%) in Ibutilide group, respectively. LA anterior wall line ablation was underwent in 2 patients (4%) in DCC group and 3 patients (6.3%) in Ibutilide group, and their block rates were 1/2 (50%) and 2/3 (66.7%), respectively. The success rate of LA BOX ablation was 2/3 (66.7%) in SCV group, 10/13 (76.9%) in DCC group, and 11/13 (84.6%) in Ibutilide group. Cavotricuspid isthmus ablation was fulfilled in 6 patients (50%) in SCV group, 10 patients (20%) in DCC group, and 15 patients (31.3%) in Ibutilide group, all of which achieved bidirectional conduction block. The rate of bidirectional conduction block of mitral isthmus line was 1/2 (50%) in SCV group, 3/5 (60%) in DCC group, and 6/8 (75%) in Ibutilide group. Superior vena cava ablation was successfully completed in one patient in SCV group and one patient the DCC group. The shortest Pd was in SCV group [43 ms (IQR: 35–52)] compared with other groups (*P* = 0.027). Fluoroscopy dose (*P* = 0.028) and procedure time (*P* = 0.001) were different among the three groups.

Two patients in DCC group and 2 patients in Ibutilide groups suffered from a groin hematoma that was resolved with compression. The rate of hematoma among three groups was not different (*P* = 0.624). No other complications, such us pericardial tamponade, embolic events, atrioesophageal fistula and malignant arrhythmias, happened in the patients enrolled in this study. There were no differences in the patients receiving AADs (amiodarone or propafenone) during the follow-up among the three groups.

### Cox regression analysis of AF recurrence after RFCA

The results for AF recurrence after RFCA at the end of follow-up by univariate and multivariate analysis are shown in Table 3. In univariate analysis, AF duration (*P* < 0.001), Scr (*P* < 0.001), LAD (*P* < 0.001), LAAD (*P* < 0.001), LVA (*P* < 0.001) and Pd (*P* < 0.001) were predictors of AF recurrence. Cardioversion type was also included in the multivariate analysis because it may be clinically related to AF recurrence. Ultimately, the multivariate Cox regression, indicated that cardioversion type (HR: 0.379, 95% CI: 0.212–0.678, *P* = 0.001), LAAD (HR: 1.179, 95% CI: 1.035–1.343, *P* = 0.013), LVA (HR: 1.061, 95% CI: 1.031–1.091, *P* < 0.001) and Pd (HR: 1.043, 95% CI: 1.015–1.073, *P* = 0.003) were independent risk factors for AF recurrence after RFCA. C statistic showed that the model had good discrimination of AF recurrence after RFCA (Cindex = 0.937, *P* < 0.001). Then, we performed a series of 1,000 bootstrap samples and revealed that the model discrimination was similar (bootstrap-corrected validation C-index = 0.938, *P* = 0.001). These results demonstrate that the ability of discrimination of the model is reliable. For further analysis of the relationship between the cardioversion types and AF recurrence, multivariate-adjusted Cox models were constructed. The Ibutilide group was defined as the reference. In the crude model that was not adjusted for risk factors of AF recurrence, compared with the Ibutilide group (reference = 1), the DCC group (HR: 1.99, 95% CI: 1.01–3.92, *P* = 0.049, Table 4) was associated with increased risk of AF recurrence. Finally, in the model 3 adjusted for AF duration, Scr, LAD, LAAD, LVA and Pd, the adjusted HR for AF recurrence was 3.57 (95% CI: 0.69–18.42; *P* = 0.129) in SCV group, and 4.10 (95% CI: 1.87–8.98; *P* < 0.001) in DCC group, compared to Ibutilide group (reference), respectively (Table 4).

**Table 3:** Univariate and stepwise multivariate cox hazard analysis of risk factors for AF recurrence after a single ablation procedure.

Variables	Univariate analysis		Multivariate analysis	
	HR (95% CI)	*P*-value	HR (95% CI)	*P*-value
Sex (male/female)	0.718 (0.371–1.392)	0.327	–	–
Age	1.037 (0.999–1.076)	0.059	–	–
AF duration	1.016 (1.010–1.022)	**<0** **.** **001**	1.001 (0.993–1.009)	0.824
BMI	1.008 (0.878–1.156)	0.911	–	–
Hypertension (yes/no)	1.483 (0.776–2.832)	0.233	–	–
CHD (yes/no)	1.219 (0.529–2.810)	0.641	–	–
Diabetes (yes/no)	1.017 (0.425–2.437)	0.969		
Smoking (yes/no)	1.220 (0.644–2.313)	0.542	–	–
Scr	1.043 (1.030–1.056)	**<0**.**001**	1.012 (0.995–1.029)	0.160
CRP	1.127 (0.930–1.366)	0.223	–	–
LAD	1.388 (1.260–1.530)	**<0**.**001**	1.039 (0.917–1.178)	0.545
LVEDD	0.976 (0.889–1.073)	0.618	–	–
LVEF	0.986 (0.943–1.031)	0.534	–	–
LAAOD	1.114 (0.932–1.331)	0.235	–	–
LAAD	1.392 (1.274–1.521)	**<0**.**001**	1.179 (1.035–1.343)	**0.013**
Cardioversion types	0.855 (0.538–1.358)	0.506	0.379 (0.212–0.678)	**0.001**
LVA	1.075 (1.053–1.097)	**<0**.**001**	1.061 (1.031–1.091)	**<0.001**
*P*-wave dispersion	1.062 (1.038–1.087)	**<0**.**001**	1.043 (1.015–1.073)	**0.003**

AF, atrial fibrillation; BMI, body mass index; CHD, coronary heart disease; CI, confidence interval; CRP, C-reactive protein; DCC, direct current synchronized cardioversion; HR, hazard ratio; LAAOD, left atrial appendage ostium diameter; LAAD, left atrial appendage depth; LAD, left atrial diameter; LVA, low voltage area; LVEF, left ventricular ejection fraction; Scr, serum creatinine; SCV, spontaneous conversion.

Bold values represent *P* < 0.05.

**Table 4:** The predictive value of cardioversion types for recurrence of persistent atrial fibrillation after radiofrequency catheter ablation.

	HR (95% CI)		
	Ibutilide group (*n* = 48)	SCV group (*n* = 12)	DCC group (*n* = 49)
Crude model	1	0.67 (0.15–2.95)	1.99 (1.01–3.92)
*P*-value		0.592	**0.049**
Adjusted model 1	1	1.13 (0.25–5.23)	2.86 (1.39–5.87)
*P*-value		0.873	**0.004**
Adjusted model 2	1	2.43 (0.49–12.16)	3.22 (1.53–6.79)
*P*-value		0.278	**0.002**
Adjusted model 3	1	3.57 (0.69–18.42)	4.10 (1.87–8.98)
*P*-value		0.129	**<0.001**

AF, atrial fibrillation; CI, confidence interval; DCC, direct current synchronized cardioversion; HR, hazard ratio; LAAD, left atrial appendage depth; LAD, left atrial diameter; LVA, low voltage area; Scr, serum creatinine; SCV, spontaneous conversion.

Crude model: no adjusted for risk factors of atrial fibrillation recurrence; Model 1: adjusted for AF duration and Scr; Model 2: adjusted for AF duration, Scr, LAD and LAAD; Model 3: adjusted for AF duration, Scr, LAD, LAAD, LVA and Pd.

Bold values represent *P* < 0.05.

### Atrial tachyarrhythmia-free rate and cumulative curve after the initial RFCA among the SCV group, DCC group and Ibutilide group

**Figure 4:**
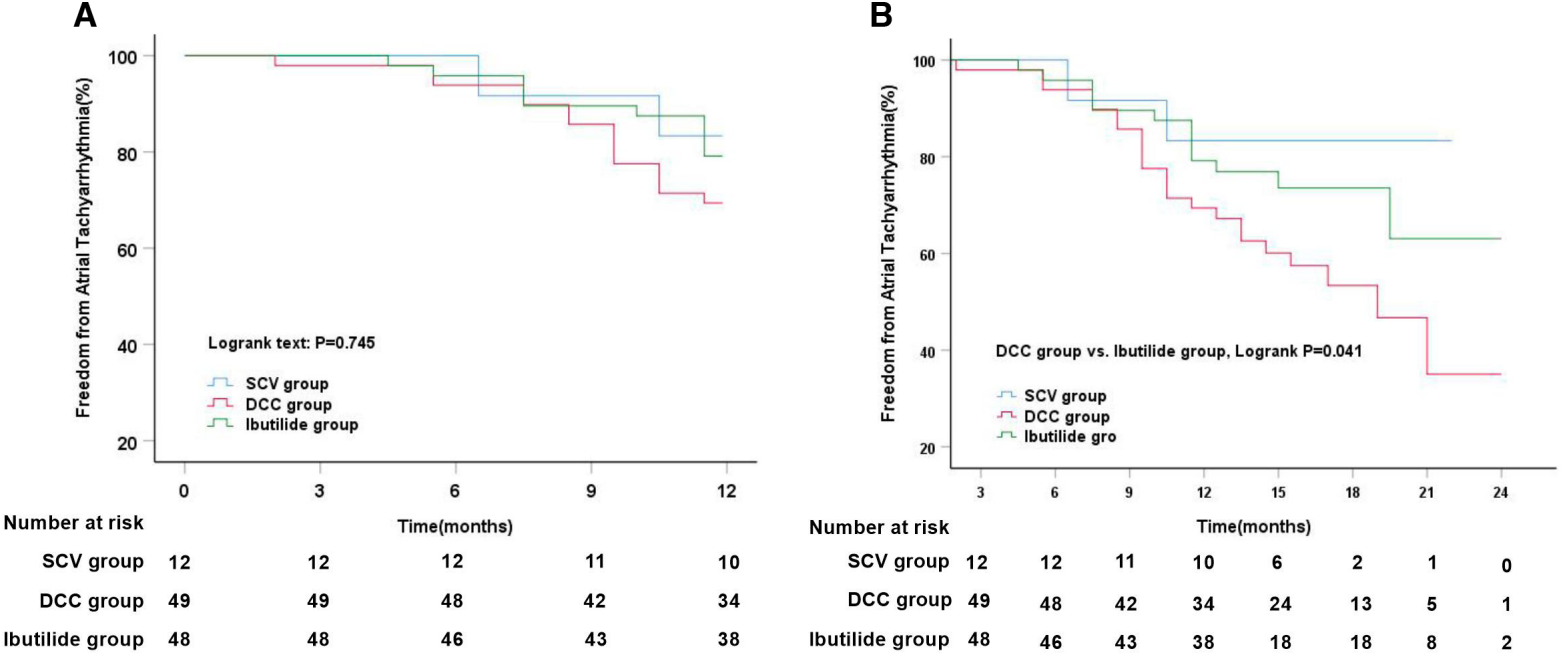
Kaplan–Meier analysis of freedom from atrial tachyarrhythmia among three different cardioversion. (**A**) Kaplan–Meier analysis of freedom from atrial tachyarrhythmia among SCV vs. DCC vs. Ibutilide groups at 12 months follow-up; (**B**) Kaplan–Meier analysis of freedom from atrial tachyarrhythmia among SCV vs. DCC vs. Ibutilide groups at median 17 months follow-up. DCC, direct current synchronized cardioversion; SCV, spontaneous conversion.

During 12-month follow-up, a slightly lower rate of freedom from ATT rates without antiarrhythmic drugs was observed in DCC group (69.4%), compared to that in SCV group (83.3%) and Ibutilide group (79.2%), but no statistical significance were reached among the three groups. Meanwhile, Kaplan–Meier analysis also showed that there was no markedly difference in the rate of freedom from ATT among the three groups (Log-rank, *P* = 0.745, Figure 4A). At the end of follow-up, the median duration of follow-up was 17 months (IQR: 15–19). However, it is distinct from the outcomes at the 12-month follow-up that the rate of freedom from ATT in Ibutilide group (72.9%) and SCV group (83.3%) was higher than that in the DCC group (53.1%) (*P* = 0.042). Simultaneously, Kaplan–Meier analysis also showed a significantly higher SR maintenance in Ibutilide group than that in DCC group (Log-rank, *P* = 0.041, Figure 4B).

### Subgroup analysis in Ibutilide group

**Figure 5:**
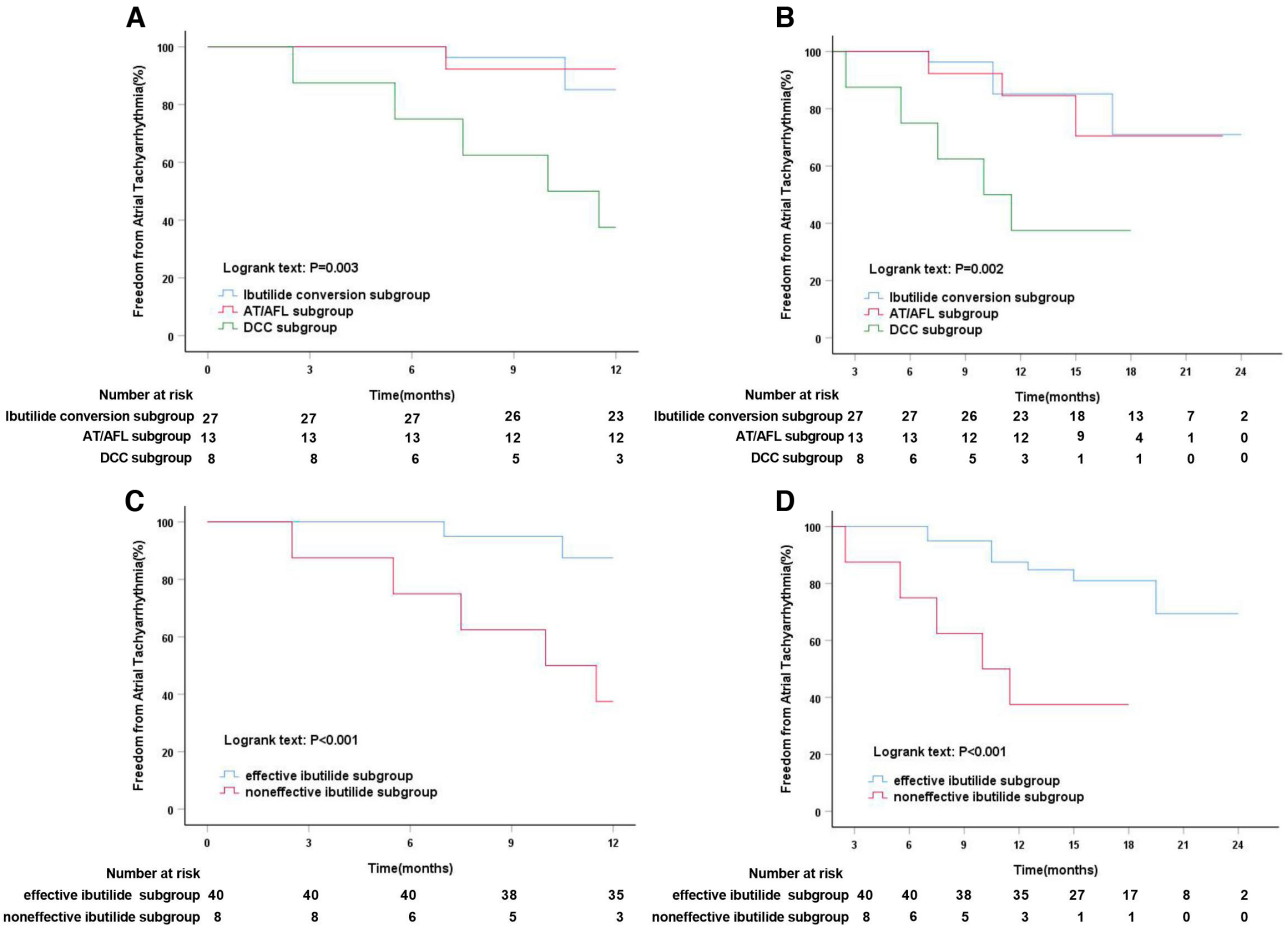
Kaplan–Meier analysis of freedom from atrial tachyarrhythmia in different Ibutilide subgroups. (**A**) Kaplan–Meier analysis of freedom from atrial tachyarrhythmia among Ibutilide conversion vs. AF/AFL vs. DCC subgroups at 12 months follow-up; (**B**) Kaplan–Meier analysis of freedom from atrial tachyarrhythmia among Ibutilide conversion vs. AF/AFL vs. DCC subgroups at median 17 months follow-up; (**C**) Kaplan–Meier analysis of freedom from atrial tachyarrhythmia in effective ibutilide vs. noneffective ibutilide subgroups at 12 months follow-up; (**D**) Kaplan–Meier analysis of freedom from atrial tachyarrhythmia in effective ibutilide vs. noneffective ibutilide subgroups at median 17 months follow-up. AFL, atrial flutter; AT, atrial tachycardia; DCC, direct current synchronized cardioversion.

After intravenous administration of Ibutilide, Ibutilide group was subdivided into three subgroups, Ibutilide conversion subgroup, AT/AFL subgroup, and DCC subgroup. Subgroup analysis showed that the event-free rate for ATT recurrence was significantly higher in Ibutilide conversion subgroup (85.2%) and AT/AFL subgroup (92.3%) than that in DCC subgroup (37.5%, Log-rank, *P* = 0.003, Figure 5A) at 1-year follow up. After a median follow-up of 17 months, the freedom from ATT rate was also significantly higher in Ibutilide conversion subgroup (81.5%) and AT/AFL subgroup (76.9%) than that in DCC subgroup (37.5%, Log-rank, *P* = 0.002, Figure 5B). By analyzing the clinical data of the three subgroups, we found that DCC subgroup had larger LAD [48 mm (IQR: 45–48), *P* = 0.001], LVA [44.5% (IQR: 26–50), *P* = 0.001], and LAAD [32 mm (IQR: 30–34), *P* = 0.014] than the other two subgroups (Table 5). According to effectiveness of Ibutilide, Ibutilide group was further subdivided into two subgroups including effective Ibutilide subgroup (AF terminated as SR or AT/AFL) and non-effective Ibutilide subgroup (AF not terminated). Kaplan–Meier analysis showed a remarkably elevated rate of SR maintenance in effective Ibutilide subgroup, compared to non-effective Ibutilide subgroup at 1-year follow-up (87.5% vs. 37.5%, Log-rank *P* < 0.001, Figure 5C) and after a median follow-up of 17 months (80.0% vs. 37.5%, Log-rank *P* < 0.001, Figure 5D).

**Table 5:** Comparison of clinical characteristics among the three subgroups.

Variable	Ibutilide group (*n* = 48)			*P*-value
	Ibutilide conversion subgroup (*n* = 27)	AT/AFL subgroup (*n* = 13)	DCC subgroup (*n* = 8)	
Age (year)	61 (54, 67)	55 (53, 65)	62 (57, 64)	0.565
Male sex, *n* (%)	16 (59.3)	9 (69.2)	6 (75)	0.652
AF duration (month)	20 (15, 30)	18 (12, 36)	48 (19, 81)	0.132
Serum creatinine (umol/L)	69 (59, 78)	75 (66, 92)	78 (57, 99)	0.455
LAD (mm)	39 (38, 43)^b^	43 (40, 46)^b^	48 (45, 49)	**0** **.** **001**
LVEF (%)	63 (58, 67)	64 (56, 66)	62 (59, 67)	0.988
LAAOD (mm)	25 (24, 26)	25 (24, 25)	26 (25, 27)	0.531
LAAD (mm)	26 (24, 29)^b^	26 (24, 30)^b^	32 (30, 34)	**0**.**014**
LVA (%)	8 (0, 12)^b^	7 (5, 41)^b^	44.5 (26, 50)	**0**.**001**
*P*-wave dispersion (ms)	56 (45, 67)	65 (45, 66)	70 (59, 78)	0.233
Complications, *n* (%)	1 (3.7)	0 (0)	0 (0)	1.000
Freedom from ATT at 1 year follow-up	23 (85.2)^b^	12 (92.3)^b^	3 (37.5)	**0**.**010**
Freedom from ATT at the end of follow-up	22 (81.5)^b^	10 (76.9)	3 (37.5)	**0**.**045**

AF, atrial fibrillation; AFL, atrial flutter; AT, atrial tachycardia; ATT, atrial tachyarrhythmia; DCC, direct current synchronized cardioversion; LAAOD, left atrial appendage ostium diameter; LAAD, left atrial appendage depth; LAD, left atrial diameter; LVA, low voltage area; LVEF, left ventricular ejection fraction.

Bold values represent *P* < 0.05.

^a^ Post hoc comparison *P* < 0.05 vs. AT/AFL subgroup.

^b^ Post hoc comparison *P* < 0.05 vs. DCC subgroup; Values are given as median (interquartile range) or *n* (%).

## Discussion

In the present study, we propose four main findings: (1) The HPSD-AI guided individualized ablation strategy, including PVI plus LA roof line ablation and personalized linear ablation based on LVZs design for patients with PsAF, are safe and effective. (2) Ibutilide-guided cardioversion is noninferior to SCV but superior to conventional DCC in maintaining SR after index ablation after a median of follow-up 17 months. (3) In patients with PsAF undergoing RFCA, Ibutilide-effective therapy significantly reduced the risk of AF recurrences compared to Ibutilide-noneffective treatment. (4) The cardioversion types including SCV, ibutilide cardioversion or DCC was independently associated with AF recurrence after RFCA.

### Catheter ablation strategy of persistent AF

PVI is the cornerstone of AF catheter ablation, particularly for paroxysmal AF (4). However, PsAF represents a major challenge in catheter ablation of arrhythmias. In recent years, randomized controlled trials have shown that additional strategies adjunctive to PVI, such as CFAE ablation, linear ablation, and the stepwise approach aiming to terminate AF, are promising to improve the clinical efficacy, but not superior to PVI alone in PsAF (5, 6, 23). In addition, significantly poor atrial substrate has been regarded as a reason for the low efficacy of the treatment in PsAF. Thus, substrate-based individualized ablation, as a novel ablation strategy, has been gradually became a hot topic in the intervention for PsAF. Unfortunately, STABLE-SR trial (24) found that the rate of freedom from ATT in PVI plus SR substrate modification at 18 months follow-up was not better than that of the conventional STEPWISE ablation approach, and STABLE-SR-II trial (8) further found that atrial-arrhythmia-free survival at 18 months follow-up was not significantly different between PVI plus substrate ablation strategy and PVI alone ablation. In summary, the optimal ablation strategy for PsAF remains poorly defined beyond PVI.

In this study, we also implemented the concept of an individualized ablation strategy for patients with PsAF. For example, PVI plus LA roof line ablation was followed by a personalized linear ablation designed based on LVZs. The results showed that LVZs was predominantly identified from LA posterior wall. Therefore, the LA BOX ablation was the primarily additional line ablation in our study. Studies have indicated that the LA posterior wall serves as one of the most key regions in pathophysiology of PsAF and is the most common non-PV site that contains AF re-entry drivers (25, 26). Furthermore, some studies have suggested that posterior wall ablation may improve the outcomes of ablation of PsAF (27, 28). Therefore, the individualized ablation strategy in our study is feasible.

### HPSD-AI ablation

Nontransmural tissue injury and discontinuity of ablation path are the pathological basis of AF recurrence after RFCA. The reason why additional ablation strategies adjunctive to PVI failed to significantly improve ablation outcomes in patients with PsAF is partly due to PV reconnection and gap-related ATT caused by extensive ablation (29, 30). Methods to improve contiguity and transmurality of atrial linear lesions may decrease the risk of AF recurrence after RFCA. AI-guided ablation strategies, including contact force, power, and time in a weighted formula have been validated to assess both contiguity and depth of lesion, and to improve clinical outcomes (13, 31). In parallel, novel approaches to apply HPSD to improve safety and create more durable lesions have been reported (32, 33). Our previous meta-analysis showed that HPSD guided by AI or lesion size index (LSI) was correlated with shorter ablation duration, higher first-pass PVI, absence of increased esophageal injury, and possibly increase freedom from AF recurrence at short-term follow-up (about 6 months), compared to low power longer duration ablation (15). In contrast, contact force sensing catheters with HPSD-AI for improving the durability of block lines were not used in three recent randomized controlled trials described above (5, 6, 23), which might be one of possible reasons for explaining the unsatisfactory overall success rate in PsAF. In our study, first-pass PVI rate under HPSD-AI ablation was 82.7%, and the bidirectional block rate of LA roof line, mitral isthmus, and posterior wall were 94.5%, 66.7%, and 79.3%, respectively. In addition, the rate of freedom from ATT at 1-year follow-up and after a median follow-up of 17 months was 75.2% and 65.1%, respectively. The similar results were reported in another HPSD-AI ablation study for PsAF (16). These evidences indicate that HPSD-AI ablation may improve clinical outcomes of treatmet for PsAF. However, the mult-icenter randomized controlled trials are needed to further evaluate the long-term outcomes of HPSD-AI ablation in the patients with PsAF.

### The Ibutilide-guided cardioversion

Interruption of the maintenance mechanisms for PsAF with atrial arrhythmogenic substrates and triggers is the key to improve the success rate of ablation. However, it is very difficult to find exact mechanisms for maintenance of AF during RFCA. Therefore, the majority of patients with PsAF needs DCC or pharmacological cardioversion, even if PVI and extra ablation are completed. Ibutilide exerts a class III antiarrhythmic effect through blocking potassium (IKr/HERG) channels and activating slow delayed inward sodium current that emerges in early repolarisation, and is commonly used for AF termination (34). The MAGIC-AF Study demonstrated that Ibutilide may reduce the LA surface area with CFAE sites, but did not prove that the Ibutilide-guided CFAE ablation strategy could improve freedom from atrial arrhythmias at 12 months for the patients with PsAF compared with PVL alone (17). A further subgroup trial suggested that AF termination by Ibutilide administration during PVI may be an appropriate strategy to minimize the ablation lesions in the patients with PsAF (35).

Our study was different from the previous two studies (17, 35). We used Ibutilide as a tool to identify the obscured critical sites in the patients who failed to restore SR after an individualized ablation strategy, and then performed targeted ablation to terminate AF instead of guiding CFAE ablation. After a median follow-up of 17 months, the freedom from ATT rate in Ibutilide group was not inferior to that in SCV group, but better than that in DCC group. Additionally, cardioversion type including SCV, Ibutilide cardioversion or DCC was independent risk factor of AF recurrence after RFCA in our study. Moreover, DCC group had a substantially highrisk of AF recurrence compared to Ibutilide group. In addition, subgroup analysis revealed that the effective Ibutilide subgroup had smaller LAD, LVA and LAAD compared with the non-effective Ibutilide subgroup, suggesting that atrial remodeling in the patients with effective Ibutilide administration may not be advanced. Moreover, studies have reported that LAD and LVA are independent risk factors for AF recurrence after RFCA (36, 37). Therefore, it can be explained that the rate of freedom from ATT was appreciably enhanced in the effective Ibutilide subgroup, compared to the non-effective Ibutilide subgroup. There are two main reasons why Ibutilide-guided cardioversion can improve freedom from AF recurrence after RFCA. On the one hand, unmasked AT or AFL may serve as a key factor in maintaining of AF after increasing the effective refractory period by Ibutilide, and targeted ablation for such sites might segregate potential triggers and interfere with electropathological substrates of PsAF. On the other hand, Ibutilide may help to determine the patients with less advanced electrical and structural remodeling, to avoid the gaps caused by excessive ablation. In a word, the individualized ablation strategy basing on the guidance of HPSD-AI and Ibutilide-guided cardioversion, are safe and effective for treatment for PsAF in our study.

### Limitations

Several limitations should be mentioned. First, this is a non-randomized and observational study with a small sample size, especially in Ibutilide subgroup, which may engender the potential to bias to our results. Therefore, our findings may not be generalized to all the patients with PsAF. Second, the rate of AF recurrence might have been underestimated because some asymptomatic patients did not see a doctor in time to complete an ECG or Holter. Third, the homogeneity of different surgeons could not be guaranteed, which may cause a potential influence on the effect of ablation. Fourth, although there was no difference in the median follow-up time among the three different cardioversion groups, the undemanding follow-up protocol after 12 months may lead to a bias of the results. Fifth, there is no consistent standards for constructing LA voltage mapping under SR or AF (8, 19–21, 23), but the voltage mapping under AF may more accurately reflect the triggering substrates of PsAF. Sixth, AF duration in some asymptomatic patients with PsAF cannot be evaluated accurately, so that the acquisition of AF duration may be subjective. Therefore, randomized multi-center trials with larger sample sizes, long-term follow-up, and rigorous protocols with wearable ECG monitoring device are needed to obtaining conclusive evidences in further.

## Conclusions

In the treatment of PsAF, Ibutilide-guided cardioversion after individualized RFCA is safe, and provides a benefit for maintaining of SR compared to conventional DCC, especially in the patients administrated with ibulide-effective therapy. This may be because Ibutilide may identify the PsAF patients with less advanced structural and electrical remodeling, and help to uncover the obscured critical sites maintaining AF followed by targeted ablation to terminate AF.

## Data Availability

The original contributions to this study has been included in the article, and further inquiries can be directed to the corresponding author.
